# Transport pathway of the Ag^+^ following artificial precipitation enhancement activities

**DOI:** 10.1016/j.heliyon.2024.e25299

**Published:** 2024-01-26

**Authors:** Xiaoyu Ren, Yongli Jin

**Affiliations:** Beijing Weather Modification Center, Beijing, 100089, China

**Keywords:** Ag^+^ transportation, Artificial precipitation enhancement, Ag^+^ accumulation, Underlying surface

## Abstract

Artificial precipitation enhancement (APE) activities have been applied extensively around the world to enhance water resources. However, the transport way of the silver iodide catalyst utilized remains completely unknown. To address this issue, in this study, we monitored the content of silver ions (Ag^+^) in a water body under the influence of APE for a period of 16 years (2004–2019). Additionally, we monitored the content of silver ions in the multi-period rainfall and soil. Our findings indicate that after the APE operation, the detected silver content in the precipitation initially demonstrated an upward trend and then decreased to 0. Furthermore, we observed that some of the silver ions remained in the air for a period extending from the time of artificial rain till the next rain. The silver ion content in the soil during the flood season was elevated by 44 % in comparison to the non-operation period; the concentration of silver ions in the water body during the operation period was 42.86 % higher than that in the non-operation period. During the long-term study, spanning 16 years, the water body played a leading role in regulating the content of silver ions released by the APE, resulting in an increase in silver ion content by 3.3 %. Our results revealed the presence of silver in the precipitation after the APE operation, indicating that silver iodide initially entered the precipitation after catalysis. Furthermore, upon the comparison of the soil and surface water during the operation period and non-operation period, the silver content during the operation period was observed to be higher than that in the non-operation period, indicating that silver iodide was incorporated into the underlying surface from the precipitation. Therefore, we have concluded that the transport pathway of silver involves its initial entry into precipitation after sowing, subsequently descending with the precipitation to reach the soil and surface water. The findings of this study establish a scale ruler for the impact of increasing global APE activities on the environment, as well as first-hand data for preventing possible future environmental risks.

## 1 Introduction

An increasing global population and the overexploitation of groundwater resources have augmented the competition for water resources, and climate change has exacerbated this competition. Hence, the redistribution of water resources artificially has become an essential research topic. Precipitation enhancement has been an effective way to utilize water resources in the air, with the history of global artificial precipitation enhancement (APE) operations starting more than 90 years ago. The principle of precipitation enhancement is to make the water in the clouds turn into raindrops and descend by applying catalysts to the local atmospheric cloud layer through aeroplanes, anti-aircraft guns, and rockets. Dry ice and silver iodide which are the two main catalysts have been used for the precipitation enhancement worldwide [1,2]. Among them, silver iodide has been the most widely used catalyst and exhibits the longest duration. In 1930, Verart from the Netherlands conducted a large-scale artificial precipitation experiment by spreading about 1.5 t dry ice at an altitude of 2500 m and induced precipitation over an area of approximately 8 km^2^ [3]. Modern weather modification activities began in 1946 with Shaefer and Vonnegut [4,5]. Schaffer successfully carried out an experiment using a plane to spread dry ice in cold clouds to form ice crystals and snow streamers. Vonnegut succeeded in the ice formation test by selecting silver iodide crystals as the artificial ice nuclei. He played a leading role in studying the method of silver iodide soot generation, revealing silver iodide to be an effective catalyst that can be successfully applied to artificial weather quickly [6,7].

Since then, large-scale artificial weather experiments have been carried out internationally. At present, the United States, Israel, China, Russia, and other countries that carry out APE mostly use aircraft operations and ground-burning flame agent operations, primarily catalyzing terrain clouds, convective clouds, and stratified mixed clouds. The United States began large-scale commercial artificial rainfall operations in the 1950s and then carried out several strictly designed artificial rainfall scientific experiments. The well-known experiments include White top Project, Climax Project, Great Lakes Project, Cyclonus Project, and the Colorado River Basin Cloud Sowing Pilot Project [[8], [9], [10], [11], [12], [13], [14], [15], [16]]. Precipitation enhancement in China began in 1958 [17,18], has after which, operations at multiple places have been carried out, successfully solving the problems associated with local meteorological droughts and extreme weather [[19], [20], [21], [22]]. In 2016, China prepared to launch the world's largest meteorological control project, "The Tianhe Project", to solve the situation of surface water shortage in northern China. The basic technical method adopted in this project was APE. In 2018, more than 30 regions in China carried out weather modification operations, using 70 aircraft sorties and 18,057 anti-aircraft artillery shells and rocket operations. The target area of precipitation enhancement operations could reach 490*10^4^km^2^, and the operation area of hail suppression could affect 53*10^4^km^2^ [23].

Over the years, the question of how to improve the effect of precipitation has been the focus of APE, but it has also masked a series of possible environmental problems [19,24]. One of them is the environmental impact of silver iodide catalysts released by continuous APE. Existing studies believe that low concentrations of silver ions are harmless to the environment and human body, but high concentrations of silver ions are toxic and will accumulate in mammalian organs [[25], [26], [27], [28], [29], [30], [31], [32]]. Before studying this problem, we should understand the transport way of the catalyst in the environment, to further discuss whether the silver iodide sown by APE will cause harmful effects on the environment. However, most studies are biased towards the change of silver ion content in a single environment, such as the monitoring of the silver ion content changes in precipitation, soil or surface water after APE operations. For instance, Warburton et al. [33,34] collected rain and snow samples in eastern Australia in 1965 after conducting an aircraft artificial rain and snow augmentation operation. Snow samples were also collected after artificial snowfall in northern Colorado and Nevada [[35], [36], [37], [38]]. In 2015 and 2016, Idaho Energy conducted snow enhancement catalytic operations in Idaho and collected snow samples [39]. Precipitation samples were collected after a catalytic operation in Hunan Province from 1978 to 1980 [40], Fujian Province from 1975 to 1986 [41], and Beijing from 2002 to 2003 [30]. Ag^+^ monitoring has been conducted in more than 100 mountain lakes and rivers in Nevada, USA since 1980 [42,43]. Zhao et al. [44,45] sampled the surface water in Beijing during the flood season from June 2004 to July 2005. In Hebei Province, dust and soil were sampled from the affected area and the control area [46]. Several other studies that conducted precipitation and surface water sampling after hail suppression also confirmed that the silver content of the samples after silver iodide seeding was higher than that before seeding or in the comparison area [[47], [48], [49], [50], [51], [52], [53]]. However, these studies did not focus on the transport pathway of silver iodide catalyst after seeding. Therefore, the objective of this study is to explain the transport pathway of the silver iodide catalyst to other components of the environment.

## **2** Materials and methods

### 2.1 Study area

**Fig. 1:**
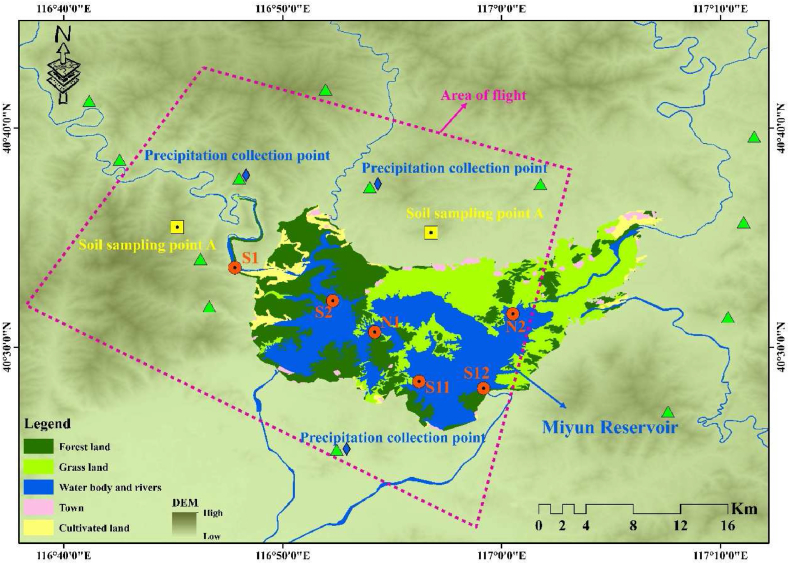
The study area and the sample points for water body, soil, and precipitation. The magenta circles represent the water sample points. The green triangles represent the rocket points for the APE. (For interpretation of the references to color in this figure legend, the reader is referred to the Web version of this article.)

The Miyun Reservoir watershed (Fig. 1) was selected for this study because it is the biggest surface drinking water source for Beijing—the megacity in China which faces a serious water shortage. During the development of the city, its water supply system depended extensively on this reservoir. Hence, the water quality of this source is highly important to the people in this city. The Miyun Reservoir is located in the North of Beijing and is 100 km away from the core area. The geographical coordinates range from 40°19′ to 41°31′N and 115°25′ to 117°33′E. The average storage capacity is approximately 43.75 × 10^8^ m^3^. This area has a temperate continental climate, summer is its primary season for rainfall and precipitation durin the winter is scarce. From 1999, the precipitation in this area has been decreasing continuously leading to a significant decline in water storage. To resolve the water shortage challenge faced by Beijing and increase the precipitation in this area, several APEs have been conducted since 2004, continuously for 16 years. Concerned about the climate, the APE was conducted primarily from May to September each year, and the platforms of APE include special aeroplanes e and APE Rockets. The performance of APE in this area was established to be satisfactory, based on the estimation conducted by the hydrology and meteorological department, from 2004 to 2019 (May to September). The APE increased the amount of water by 21,261 × 10^4^ m^3^ in the reservoir, which accounted for 8.63 % of the total input water of the Miyun Reservoir.

In this research, in-situ experiments, including estimation of the quality of water and soil samples, as well as precipitation monitoring for silver ions were conducted. Water sampling in the Miyun Reservoir was conducted over a period of 16 years since the beginning of APE in 2004. Soil sampling and precipitation monitoring for silver ion concentration continued continuously over the last two years since 2018. There were six samples, three operations, and two mountain points set for water sampling, precipitation monitoring, and soil sampling, respectively (Fig. 1). Due to the climate in this area, the rainfall occurred exclusively from June to September. APE activities were conducted frequently in these months and this was called the operation period (OP), the period from October to April of the following year was called the non-operational period (NOP).

### 2.2 Method of sample collection

#### 2.2.1 Precipitation sample collection

In the areas affected by APE, the operation shots were selected as precipitation collection points to study the migration process of silver ions in the precipitation. When precipitation occurs, we use a deionized bucket to collect rainwater, and subsequently pour it into a rainwater collector and store it in the dark. Effective collection meant that there was a precipitation process and the precipitation collection volume was greater than or equal to 500 ml. The collected precipitation samples were tested for their silver ion content.

#### 2.2.2 Soil sampling method of Ag^+^ ions in the soil

To comprehensively study the transport pathway of silver ions, two locations (Yanjiaping and a coffee shop) for soil sampling were designated around the Miyun Reservoir (Fig. 1). Soil in these two locations were deemed to be influenced by the APE and soil samples were collected at two-time points in the beginning and the end of the operation period in early June and early September. Initially, the location information of soil samples was recorded. The sampling principle was adopted according to the slope type. Plants and humus layer covering the surface soil were removed, and then the soil sample extending from the surface to the parent material layer was collected. For every 10 cm about 500 g of soil sample was collected, and placed in plastic bag. The repeated sampling was conducted within 1 m of the previous sampling location. The location information of the soil samples was recorded before sending the samples to a laboratory for testing. Subsequently, the silver ion content was determined d in the laboratory.

#### 2.2.3 Water body sampling of the reservoir

Since June 2004, our research group began to sample the water in the Miyun Reservoir. To get complete detailed information about the Ag^+^ concentration, a linear sampling method which included six points that ranged from the upstream to the exit of the reservoir was set (Fig. 1). The sampling points were located away from the area of human activity, which reduced the influence of the human beings on the data. The frequency of the sampling was twice a month and the dates were finalized and set before and after the fifth day of each month. Water samples were collected from the reservoir by using a vertical water picker located 30 cm under the water's surface. Subsequently, the samples were placed in a bottle cleaned with distilled water. After the water at all of the points were collected they were then transported to the laboratory directly.

### 2.3 Method employed for determining the Ag ^+^ values

#### 2.3.1 Laboratory chemical method to elucidate the concentration of the Ag^+^ in water

After the water samples were collected, we tested the collected samples. The detection basis of silver ions employed “Standard Test Method for Drinking Water, Metal Index GB/T 5750.6-2006 1.5”. The silver ion analysis method adopted inductively coupled plasma mass spectrometry. The testing protocol included the following steps: 1) Seven 25 ml colorimetric tubes were taken and the following volumes of silver standard solution were added: 0 ml, 0.20 ml, 0.40 ml, 0.60 ml, 0.80 ml, 1.00 ml and 2.00 ml. This was followed by adding 5 ml hydrochloric acid solution to each tube. 2) Subsequently, add 2.5 ml sodium hydroxide solution, 1.0 ml potassium periodate solution, and 0.5 ml potassium persulfate solution were added to the sample and standard tube, which was followed by diluting this solution to a final volume of 25 ml with pure water. Subsequently, the bottles were shaken and placed in a boiling water bath immediately, followed by heating for 20min, and cooled at room temperature. 3) The absorbance of the samples, using pure water as a reference, was then measured at 355 nm wavelength, using a 3 cm cuvette. 4) A standard curve was then plotted and the quatity of silver in the sample tube was evaluated.

The computation formula employed for determining the mass concentration of silver in the water sample is as follows:

(1) $$\rho \left(Ag\right)=\frac{m}{V}$$

where ρ(Ag) represents the mass concentration of silver in the water sample, in milligrams per litre (mg/L); m represent the mass of silver in the water sample determined using the standard curve, in micrograms (μg); and V represents the water sample volume, in millilitres (mL).

#### 2.3.2 Laboratory chemical method to elucidate the concentration of the Ag^+^ in soil

After the soil samples were collected, we tested the collected samples. The protocol included the following steps: 1) Pretreatment: using 12 mL nitric acid and 4 mL hydrofluoric acid to digest 0.2 g of soil sample. 2) Starting the mass spectrometer and using the mass spectrometer tuning solution to perform the mass calibration and resolution check, and verifying the calibration. 3) Before sample analysis, the system was flushed with 1–2 % HNO_3_ solution. 4) Each of the samples was then nebulized until a steady-state signal was obtained. 5) Data were collected and the CCV (Continuous Calibration Verification), LLCV (lower limit Calibration Verification), and CCB (Continuous Calibration Blank) for every 10 samples were analyzed.

After the digested sample was weighed, we immediately weighed another 5–10 g of the sample that was closest to 0.01 g and placed it into a tared crucible. We dried this aliquot at 105 °C and then allowed it to cool in a desiccator before weighing it, and then calculated the dry weight%.

(2) $$\text{dry}\;\text{weight}\%=\frac{\text{dry}\;\text{sample}\left(g\right)}{\text{sample}\left(g\right)}\times 100$$

Calculating the Ag^+^ concentration:

(3) $$\text{concentration}\;\text{DW}=\frac{C\times V}{W\times S}$$

where the DW represents v the concentration based on dry weight (mg/kg); C represents the digestion concentration (mg/L); V represents s the final volume after sample preparation (L); W represents the wet sample weight (kg); and S represents the % Solid content/100 = % dry weight/100.

## 3 Results

### 3.1 Ag^+^ from the APE did not descend to the ground immediately

**Fig. 2:**
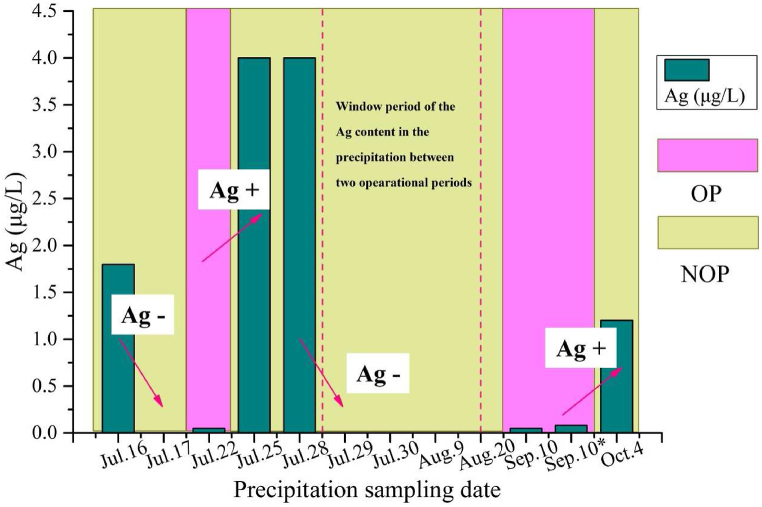
The Ag ^+^ concentration in the precipitation during the operation period (OP) and non-operation period (NOP).

From June to October 2019, the precipitation collections were performed during the APE and the intermissions, totalling 12 instances (Fig. 2). The results indicated that the Ag ^**+**^ concentration in the rainfall was higher than that in the reservoir. Meanwhile, when the APE was being performed, the Ag^+^ concentration in the rainfall was significantly low; but this value became substantially high in the first or second rainfall in the APE intermission.

For instance, the Ag^+^ concentration in the rainfall reached 1.8 μg L^−1^ on June 16 after the APE, while this value was zero in the rainfall on June 17. On 22 July, the day when APE was performed, the Ag^+^ concentration in the rainfall was merely 0.05 μg L^−1^, but this value increased to 4.0 μg L^−1^ in the first two rainfalls after the APE. Subsequently, in the next four rainfall events, the Ag^+^ concentration was lower than 0.03 μg L^−1^. This also happened on September 10, when two APE activities were performed, the Ag^+^ concentration in the rainfall was 0.05 μg L^−1^ and 0.07 μg L^−1^, but this value reached 1.2 μg L^−1^ in the first rainfall after the APE on October 4.

This may be because the Ag^+^ from the APE did not act as the ice nucleus of the rainfall itself but merely as a catalyst to produce the hydrometeor containing Ag^+^ to enhance the formation of the raindrop. Hence, in the precipitation when the APE was being carried the Ag^+^ concentration was low and did not descend with the precipitation immediately. Ag^+^ floating in the upper atmosphere may have descended with the precipitation in the APE intermission through wet deposition. This meant an increase in the Ag^+^ concentration in the following rainfall after the APE. There was an Ag^+^ window between the two operational periods, where that the Ag^+^ concentration in the precipitation was very low, for instance from July 29 to October 20 (Fig. 2). It can also be observed from Fig. 2 that when all the silver ions from APE descended to the ground with the precipitation, the silver ion content in the precipitation decreased to 0. In other words, the content of silver ions in catalytic precipitation is higher than that in natural precipitation. This indicates that the silver iodide catalyst sown by APE enters the precipitation, which is the first stage of the transport pathway of silver ions.

Compared with other countries and regions, the silver content of the precipitation in this study ranged from 0 to 4 μg L^−1^, which is higher than the silver content data values in Australia (1965) and Idaho (2015–2016) [33,34,39]. When compared with the silver content of catalytic precipitations in other regions of China (Table 1), the silver content of catalytic precipitation in Beijing in 2019 was higher than that of the 496 precipitation samples collected in Hunan Province during 1978–1980 [40], 186 precipitation samples collected in Beijing during 2022–2023 [30], and the silver content in 183 precipitation samples collected in Qinghai Province in 2023. However, it is lower than the silver content in the 194 precipitation samples collected by Zhao et al. in 2002–2003. Although the silver content of the catalytic precipitations in this study reached 4 μg L^−1^, it was still lower than the Chinese drinking water quality and sanitation standard of 50 μg L^−1^. Therefore, this study concluded that the silver iodide catalyst sown by artificial rainfall activities had no effect on the precipitation, and it also indicated that the silver iodide catalyst sown by APE activities initially entered the precipitation. The difference in silver content in the catalytic precipitations in the Beijing area may be related to the amount of catalyst, the setting of sampling points, the mode of catalysis and the form of circulation.

**Table 1:** The silver content in artificial precipitation in different countries or regions.

Country ＆ Region	Catalytic time/(year)	Number of samples	Silver content/(μg·L^−1^)
Australia	1965	63	0.003–0.048
Idaho, USA	2015–2016	4000	0.005–0.08
Hunan Province, China	1978–1980	496	0.005–0.65
Beijing, China	2002–2003	194	0.067–7.46
Beijing, China	2022–2023	186	0.004–1.63
Qinghai Province, China	2023	183	0.04–2.4

### 3.2 Soil Ag ^+^ concentration increased prominently after the APE

**Fig. 3:**
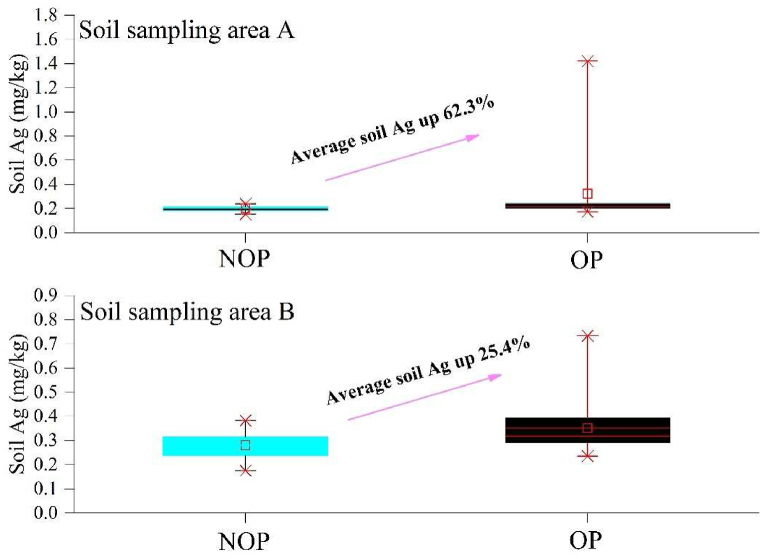
Soil Ag ^+^ concentration in the operation period (OP) and non-operation period (NOP) at the two sampling locations.

After the APE activities which included ground and plane operations, the Ag ^**+**^ content in the soil increased prominently in the two sampling points (Fig. 3). In the Yanjiaping area, where frequent APE activities were implemented from June to September, and included 78 rounds of ground operations and one plane operation, 3292 g silver iodide catalyst were used in the ground operations and 486 g were used in the plane operation. After these APE activities, the Ag ^**+**^ concentration in the soil increased to 0.35 mg kg^−1^ from 0.28 mg kg^−1^, which accounted for an increase of 25.4 %. In the Coffee Shop area, 82 rounds of ground operations and three rounds of plane operations of APE were performed from July to September. A total of 4,127g and 827g silver iodide catalysts were used in the ground operations and plane operations in the APE activities respectively. During this period, the soil Ag ^**+**^ concentration in this area increased from 0.20 mg kg^−1^ to 0.32 mg kg^−1^, which accounted for an increase of 62.3 %. This indicates that silver ions leach into the soil with precipitation, which is the second stage of the transport pathway of silver ions. In the two areas, the average soil Ag ^**+**^ concentration increase was approximately 45 % following the frequent APE activities. The soil Ag ^**+**^ exhibited a larger increase percentage in the Coffee Shop than in the Yanjiaping area due to the higher frequency of APE events being implemented in that area. This implies that APE was the direct and prominent cause of the increase in soil Ag ^**+**^ concentration. Meanwhile, the vegetation on the ground also intercepted part of the Ag^+^ amount from the APE, since the total soil Ag^+^ concentration was higher in the Yanjiaping, which has better vegetation coverage than the Coffee Shop. The increase in interception approximately ranged from 8.6 %–28.6 %.

As can be seen from Table 2, the silver content of the 124 soil samples collected from the affected areas of Hebei Province in 2011 was 0.0519–0.1010 mg kg^−1^ [46], and that of the 56 soil samples collected from Qinghai Province in 2023 was 0.0235–0.661 mg kg^−1^. The silver content of the 320 soil samples from the affected areas of APE collected in Beijing in 2023 was 0.11–1.43 mg kg^−1^. The silver content of the soil samples from the affected areas of APE collected in this study was 0.154–1.42 mg kg^−1^, which was higher than the silver content of the samples collected from Hebei Province and Qinghai Province. The possible reasons are related to the type of underlying surface, the amount of catalyst, the distribution of the sampling locations, and the surface runoff capacity. The silver content in the soil in this study was approximately equal to that in the soil samples collected in Beijing in 2023, which may be attributed to the poor fluidity of the soil. It may also indicate the risk of silver enrichment in the soil. However, due to the limited time of data collection, more data is needed to evaluate the impact of APE on the soil environment in further studies.

**Table 2:** The silver content in soil in several regions.

Country ＆ Region	Catalytic time/(year)	Number of samples	Silver content/(mg·kg^−1^)
Hebei Province China	2011	124	0.0519–0.1010
Qinghai Province, China	2023	56	0.0235–0.661
Beijing, China	2023	320	0.11–1.43

### 3.3 The influence of APE on Ag ^+^ concentration in the reservoir

#### 3.3.1 APE disrupted the original balance of the Ag^+^ concentration in the reservoir

**Fig. 4:**
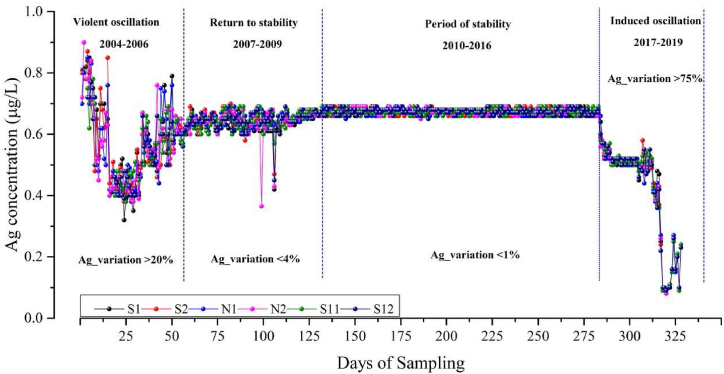
Variation of the Ag ^+^ concentration in the reservoir in the past 16 years.

From 2004 to 2019, APE activities were continuously performed every year and the Ag ^**+**^ concentration in the water displayed a prominent variation in the different periods (Fig. 4). From the beginning of the APE initiation to this day, the Ag ^**+**^ concentration experienced four fluctuations periods in the span of 16 years, which included a violent oscillation period, a return to stability period, a stable period, and an induced oscillation period. In the violent oscillation period, from 2004 to 2006, the Ag ^**+**^ concentration in the water drastically varied with a rate of change that exceeded 20 %. From the beginning of the APE, the concentration increased continuously and the highest value exceeded 0.9 μg L^−1^. Subsequently, starting from 2005, the Ag ^**+**^ concentration began to decrease and the lowest value was merely 0.32 μg L^−1^, but from 2006, the concentration increased to 0.7 μg L^−1^. This indicates that APE was the reason for the significant increase in Ag ^**+**^ content in the water at the beginning. However this increase in trend was disrupted by the external water transfer from other six reservoirs located outside of Beijing [54], which plausibly diluted the continuously increasing concentration of Ag^+^ ions. In the second period from 2007 to 2009, the amplitude of variation was smaller than in the first period, and the variation of the Ag ^**+**^ concentration became stable. The variation rate in this period was 4 %, and the average Ag ^**+**^ concentration was 0.63 μg L^−1^. From 2010 to 2016, the variation of Ag concentration in the Miyun Reservoir was very small and the Ag ^**+**^ variation rate was less than 1 %, this was called the stable period. The highest value in this period was 0.69 μg L^−1^ and the lowest value was 0.63 μg L^−1^, the average value was 0.67 μg L^−1^. From 2017 to the present date, the Ag ^**+**^ concentration has entered an oscillation period again, in which the variation rate exceeded 75 %, the highest value was 0.67 μg L^−1^ and the lowest one was 0.10 μg L^−1^. This was distinct from to the first oscillation period in which the Ag ^**+**^ concentration increased, the Ag ^**+**^ concentration in this period was observed to decrease prominently. This decrease may have been induced by the external water transfer from the South–North Water Transfer Project, which started in 2017, there were about 24,910 × 10^4^ m^3^ of water that flowed into the Miyun Reservoir from 2017 to 2019 (data from Beijing Hydrological Station). This would have prominently diluted the concentration of Ag^**+**^ within a short time interval.

#### 3.3.2 Ag^+^ concentration demonstrates a higher value in the APE operation period

**Fig. 5:**
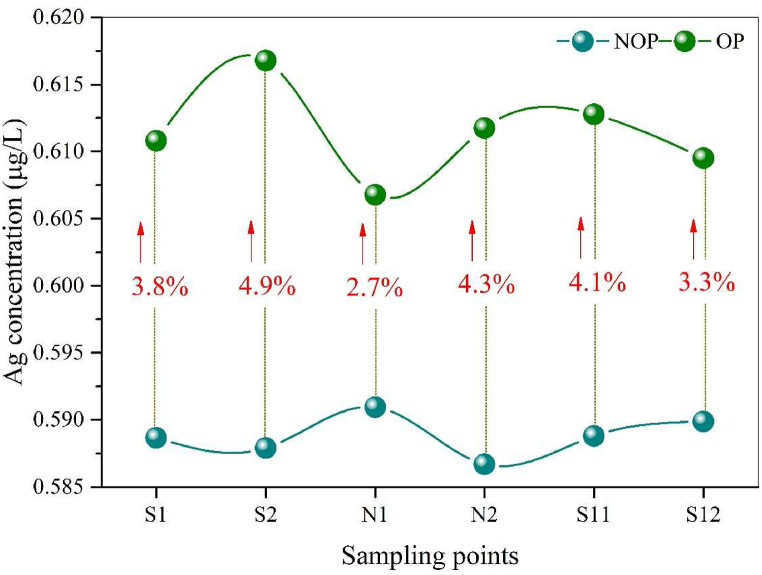
Comparison of Ag ^+^ concentration in the operation period (OP) and non-operation period (NOP) over the long term at the sampling locations.

The average Ag ^**+**^ concentration at the six water sampling locations in the OP and the NOP from 2004 to 2019 was analyzed (Fig. 5). Over the 16 years, the Ag^+^ concentration was higher in the OP than in the NOP. The content of Ag^**+**^ in the six locations differed from 2.7 to 4.9 % in the OP and NOP. Among the six locations, S2 demonstrated the highest variation rate which reached 4.9 %, and N1 demonstrated the smallest variation which was merely 2.7 %, the other four locations demonstrated similar variations. Among the six locations, N1 was at the centre of the Miyun Reservoir, the Ag^+^ concentration there exhibited the highest value in the NOP and the lowest value in the OP. This may be attributed to the fact that the fluidity of water was not as strong at this point in comparison to other points, which this decelerated the influence of APE activities. Over the long term, the APE activities resulted in an increase of the Ag ^**+**^ concentration in the OP area, and it also influenced the balance of the Ag ^**+**^ concentration in the water, but the influence was not prominent because the average variation rate of the Ag ^**+**^ concentration was merely 3.85 %. As can be seen in Fig. 5, the silver content of all samples in the OP was higher than that of NOP, indicating that after the catalytic operations of the APE, silver ions descend to the underlying surface with precipitation, and some of them directly descend into the surface of the water body, while other parts may flow into the surface water body with soil erosion, surface runoff. This accounted for the third stage of the transport pathway of silver ions.

As seen in Table 3, taking Moldova as an example, the Ag^+^ concentration in the reservoirs affected by artificial precipitation was 1.7–7.4 μg L^−1^, while the Ag^+^ concentration in the reservoirs of the comparison area was 0.9–4.1 μg L^−1^ [23,53]. The silver content data of the 495 surface water bodies collected in Beijing from 2021 to 2023 were 0.07–0.42 μg L^−1^. Among them, the silver ion content during the catalytic operations was 0.08–0.42 μg L^−1^, and the silver ion content during the non-catalytic operations was 0.07–0.12 μg L^−1^. The Ag ^+^ concentration in the affected area was higher than that in the comparison area. The results are consistent with the conclusions of this study, indicating that after seeding the silver iodide catalyst, silver ions first enter the precipitation, and then descend to the surface water with the precipitation. In this study, the Ag^+^ content in the OP phase was 0.23–0.76 μg L^−1^, and the Ag^+^ content in the NOP phase was 0.15–0.68 μg L^−1^, both of which were lower than the values in the Moldova experiment. The silver content of the 495 surface water bodies collected in Beijing from 2021 to 2023 ranged from 0.07 to 0.42 μg L^−1^, while that of Qinghai from 2023 ranged from 0.08 to 0.15 μg L^−1^, which is lower than that of the surface water samples collected in this study. Consequently, the silver content of the surface water in Beijing during 2021–2023 was lower than that observed in this study, which may be due to the decrease in the number of artificial rainfall activities during the COVID-19 pandemic, resulting in a decrease in the amount of catalyst seeding.

**Table 3:** Silver content in surface water after APE in several regions.

Country ＆ Region	Catalytic time/(year)	Number of samples	Silver content/(μg·L^−1^)
Moldova	1986–1991	1800	0.9–7.4
Beijing, China	2021–2023	495	0.07–0.42
Qinghai Province, China	2023	37	0.08–0.15

APE, artificial precipitation enhancement.

#### 3.3.3 Single APE activity prominently increases the Ag^+^ content in the water

In the short term, we investigated whether a single APE activity can influence the Ag ^**+**^ content in the water (Table 4). Two single APE operations in June and September 2019 were selected, and the average Ag ^**+**^ content was obtained at the six locations before and after the single APE activity. Form Table 4, the Ag ^**+**^ concentration increased significantly after the APE at all six sampling locations, and the average increased amplitude was 42.9 %. All the increased ratios at the sampling locations exceeded 25 %, and this means that after the APE the Ag ^**+**^ concentration increased rapidly, and the water body was like a container that held the Ag^**+**^ from the APE action for a short time.

**Table 4:** The comparison of Ag ^+^ concentration before and after the APE.

Samples	S1	S2	N1	N2	S11	S12	Average	Sum
Before OP (μg/·L^−1^)	0.07	0.06	0.07	0.07	0.06	0.08	0.07	0.41
After OP (μg/·L^−1^)	0.10	0.10	0.10	0.09	0.10	0.10	0.10	0.59
Amplitude	42.9 %	66.7 %	42.9 %	28.6 %	66.7 %	25.0 %	42.9 %	43.9 %

APE, artificial precipitation enhancement.

## 4 Discussion

### 4.1 The primary transport pathway of the Ag ^**+**^ originating from the APE activities

In this study, the content and variation of the Ag^+^ from the APE activities in the precipitation (Fig. 2), water bodies (Fig. 4, Fig. 5), and soil (Fig. 3) were obtained by the aforementioned laboratory experiments. From the results, we deduced the transport pathway of Ag^+^ after it was used for the induction of the APE. Silver ions and iodine ions were produced by photolysis of the silver iodide catalyst sown by APE. Iodine ions are required for human nutrition and they readily undergo sublimation. Hence, the transport pathway of silver ions was exclusively considered in this study. After silver ion seeding, the silver content was detected in the precipitation, indicating that the silver ions first entered the precipitation. Then, through the comparison of the silver ion content in the soil samples, it was found that the silver content in the soil changed significantly before and after APE operations, indicating that silver ions descended to the soil with precipitation. While monitoring the silver ion content in the surface water, it was observed that during OP, the silver content in all samples was higher than that during NOP, indicating that silver ions dispersed by artificial rainfall descended to the surface water with precipitation, but there were other sources of silver ions, including soil erosion and surface runoff that could have contributed to the soil silver ion levels. However, this aspect was not explored in this study. The soil and water bodies were the primary sites that retained the Ag^+^ as they were the two crucial land surface covers. The transport pathway of the Ag^+^ was mainly through precipitation, soil retention, water body transformation, and plant interception. Plant interception was not studied in this research because the ability of interception of each plant was different [55], and there are several plant types in this area. However, this study indicated that precipitation, soil, and water bodies were the three primary destinations of Ag^+^ originating from the APE. It was found that the silver ion content in the soil did not decrease with time. If there are other land use types, including residence zones, and aquaculture zones, they may face Ag^+^ accumulation risk in the future. Ag^+^ in the precipitation underwent a unique but non-elected process, as there was a time delay in this particular process (Fig. 2). This may be because Ag^+^ played the role of catalyst in the APE activity, but it did not become the condensation or sublimation nucleus immediately. The silver ions then descended with the larger raindrops. This crucial phenomenon makes Ag^+^ from the APE become pollution particles which float in the air and are moved via other weather processes, including a big cyclone. This polluted air may influence other areas where the APE activities were not conducted.

### 4.2 Water bodies retain and transfer Ag ^**+**^ originating from frequent APE activities

From the results (Fig. 4, Fig. 5, Table 4), it is evident that water bodies can retain Ag^+^ over short periods and regulate the Ag^+^ concentration over long periods by transferring water. When compared with the studies in Moldova [23] and Beijing (Table 3), the concentration of Ag^+^ in the affected area was higher than that in the comparison area, and the results were consistent with the conclusions of this study. In the OP of the APE of this study, the Ag^+^ concentration increased prominently and reached 42.86 %, but in the NOP, the Ag^+^ content in the water was minimal (Table 4). This proved that the water body had a cumulative effect when the APE happened over the short term. Two Ag^+^ sources were the main driving forces that made this possible. The first was the Ag^+^ from the precipitation which fell into the water body directly. The second one was the Ag^+^ from surface runoff and soil erosion. Ag^+^ in the runoff not only included the source from the precipitation but it was also combined with Ag^+^ in the soil that was transferred by the confluence process [56]. Soil erosion was a non-negligible process that drove Ag^+^ to leave the soil to the water body due to the topography mediated by the substantia vegetation cover.

However, over the long term, Ag^+^ concentration in the water body did not exhibit a drastic variation (Fig. 4), which implies that the water body can attenuation and regulation (Fig. 5). In this study, water in the reservoir was flowing and interchangeable with external water sources, though these processes were very slow in the natural conditions. This will keep the dynamic balance of the Ag^+^ concentration in the reservoir over the long term. At the same time, artificial water transfer to this reservoir of Beijing [54] broke the balance of Ag^+^ concentration in the natural conditions which meant that the Ag^+^ content drastically decreased (Fig. 4).

Though the Ag^+^ concentration was far below the drinking water standard, it should not be neglected that the total content of Ag^+^ was increased due to the APE activities. In the future, the Ag^+^ content will increase if the APE activities are continuously conducted.

### 4.3 Retention and accumulation of Ag ^**+**^ originating from the APE by the soil

The silver content of soil samples in this study was higher than that in Hebei Province and Qinghai Province, and higher during the operations than during the non-operations. The silver content value was almost the same as that of the soil samples collected in Beijing in 2023, which may be due to the poor soil mobility, resulting in the accumulation of catalytic silver ions in the soil. Soil exhibited a substantial ability for the retention of Ag^+^ originating from APE activities which reached 44 % (Fig. 3). The key reason is that soil can adsorb the Ag^+^ ions with the interspace on its surface and other charged ions on the soil particle surfaces [57,58]. At the same time, the accumulation of the Ag^+^ from the continuous APE activities by the soil was also crucial. Soil is a key element in the ecosystem which supplies the water, organic matter and nutrients for the plants and the living environment for animals. The pot experiment of Causapé et al. (2021) showed that the growth of wheat was limited when the silver concentration in the soil increased, and the pot with the highest concentration exhibited a lack of growth of wheat [59]. Soil is like a sponge that supplies enough space for the Ag^+^ accumulation and other ions can have a chemical reaction with Ag^+^, including the cupric ion [60,61]. The accumulation in the soil due to the continuous APE activities, will not only make the soil the “Ag^+^ bank” but also to be the “Ag source” in the ecosystem. This source will transfer the accumulated Ag^+^ to other locations (e.g. water bodies) through rainfall and soil erosion [62]. Notably, due to the chemical property of the Ag^+^ being stable, even if the APE activities are stopped, the accumulated Ag^+^ may remain in the soil over the long term and could be released into the environment by the external driving forces. The silver content in the sediment was higher than that observed by Causapé et al. [63,64] in the soil of the same area. According to Smith and Carson [57], this implies that silver is transported and lixiviated by streams. However, this study has not collected data on this aspect, therefore further exploration is required.

### 4.4 The Ag^**+**^ from the APE will cause risk to the environment and drink water source

Though the Ag^+^ concentration in the water increase by 42.86 %, this concentration was still far below the Ag^+^ content threshold of the drinking water in China. However, three risks to the environment and ecosystem which the Ag^+^ from the APE may bring should not be neglected. First, the residual Ag^+^ from the APE in the air could become a new aerosol pollutant, that could drift away to other areas by floating in the air and this may bring damage to the ecosystem of other areas where the APE was not conducted. Second, soil serves as a bank of Ag^+^ originating from APE, as extensive Ag^+^ accumulate in the soil following continuous APE activities. These Ag^+^ ions may be absorbed by plants and bring harm to their growth, Subsequently, animals would also be affected if they ate the polluted plants since Ag^+^ can accumulate in the brain [27]. Through precipitation and soil erosion, the Ag^+^ may be transferred to other places that may negatively influence to the ecosystems, specifically, agricultural ecosystem. Additionally, fungi and other microorganisms may potentially be damaged by the external Ag^+^ [[65], [66], [67]], Ag^+^ may lead to the destruction of the respiratory chain of cells [68], bind to sulfhydryl or other proteins, and inhibit the enzyme activity of bacteria involved in the phosphorus, sulfur, and nitrogen cycles [[69], [70], [71], [72], [73]], This can inactivate microorganisms [74]. At the same time, both nitrifying microorganisms and ammonia oxidation rates in soil may be negatively affected [75]. On the other hand, Ag^+^ penetrates the cell membrane into the cell interior and can inhibit cell division and destroy DNA replication [[76], [77], [78], [79], [80], [81]]. Soil microorganisms are an important component of farmland ecosystems and an important index for maintaining soil fertility and crop production [82]. Therefore, silver ions will inevitably affect soil productivity on the premise that they affect soil microorganisms. The decline of soil productivity also leads to the destruction of soil ecosystems. The Ag^+^ in the soil may combine with other ions, such as cupric ions, and then produce toxic pollutants. Meanwhile, Ag^+^ is also an important catalyst for causing other types of pollution [83]. Third, our study indicates that the water body can adjust the concentration of Ag^+^ over the long term to ensure that the content does not become very high; however these Ag^+^ may be deposited in the bottom soil of the water body. This may generate a long-term Ag^+^ release source and cause a substantial g risk to the drinking water source.

## 5 Conclusions

In-situ experiments were performed in this study to find the accumulation mechanism and the transformation process of Ag^+^ from the continuous APE activities in the drinking water source area. The Ag^+^ content in the precipitation, soil and reservoir were obtained during the continuous observation. Results showed that Ag^+^ from the APE will descend with the precipitation, but the Ag^+^ can be retained in the air for the duration between the APE and the next precipitation. The soil acted as a bank that revived more than 44 % Ag^+^ in the APE operation time than in the non-operation time. Additionally, the Ag^+^ may accumulate in the soil in the long run. The water body had a prominent ability to retain the Ag^+^ from APE at a short duration and the concentration increased by 42.86 % in the APE operation period. In the long run, Ag^+^ concentration increased by 3.3 % since the water body could move the Ag^+^ to other places. Additionally, the Ag^+^ concentration was stable if there was no artificial external water transfer. The silver content was detected in the precipitation after seeding, indicating that the silver ions entered the precipitation first. The silver content in soil samples was higher in the OP than in the NOP, indicating that the silver ions also descended to the soil with precipitation. The silver content in the surface water body was measured, and the silver content in the OP was higher than that in the NOP, indicating that the silver ions fell to the surface water body with precipitation. Therefore, the transport pathway of silver ions after seeding the silver iodide catalyst in APE, was that the silver ions first enter the precipitation, and then descend with precipitation to the underlying surface, including soil and surface water. Even with the increasing frequency of the APE activities, the silver ion content in the precipitation and underlying surface is still within the safe range. Therefore, there can be no adverse impact on the environment in the short term. However, considering the possible accumulation of sediment in soil and water bodies, more data are needed to evaluate its far-reaching impact on the environment.

## Data availability statement

The data associated with the study has not been deposited into any publicly available repository. It will be made available **on** request.

## CRediT authorship contribution statement

**Xiaoyu Ren:** Writing – review & editing, Writing – original draft, Software, Project administration, Methodology, Investigation, Formal analysis, Data curation, Conceptualization. **Yongli Jin:** Validation, Supervision, Resources, Funding acquisition.

## Declaration of competing interest

The authors declare that they have no known competing financial interests or personal relationships that could have appeared to influence the work reported in this paper.
